# Sex-specific cut-off for dilute Russell’s viper venom time lupus anticoagulant test may be of value

**DOI:** 10.1016/j.rpth.2024.102657

**Published:** 2024-12-12

**Authors:** Ning Tang, Yuanmei Luo, Mangui Li, Mingchao Zhu, Dengju Li

**Affiliations:** 1 Department of Clinical laboratory, Tongji Hospital, Tongji Medical College, Huazhong University of Science and Technology, Wuhan, China; 2 Department of Clinical laboratory, Qingyuan People's Hospital, Qingyuan, China; 3 Department of Clinical laboratory, Qinghai Red Cross Hospital, Xining, China; 4 Department of Clinical laboratory, Tianmen First People’s Hospital, Tianmen, China; 5 Department of Hematology, Tongji Hospital, Tongji Medical College, Huazhong University of Science and Technology, Wuhan, China

**Keywords:** antiphospholipid syndrome, dilute Russell’s viper venom time, lupus anticoagulant, sex-specific cut-off

## Abstract

**Background:**

Current guidelines recommend application of the 99th percentile to determine the cut-off value on at least 120 healthy donors regardless of sex for lupus anticoagulant (LA) ratio of each step. However, a statistically significant difference between the sexes has been found for LA ratio recently.

**Objectives:**

To clarify whether this sex difference in dilute Russell’s viper venom time (DRVVT) exists in various detection systems and the necessity of setting sex-specific cut-off values.

**Methods:**

Blood samples from healthy donors were detected on 3 DRVVT detection systems, and the sex-specific cut-offs of DRVVT test were obtained based on the 99th or 97.5th centile of screen, confirm, and normalized ratios (NRs) grouped by sex in each system. One thousand one hundred twenty one female patients with suspected antiphospholipid syndrome (APS) were retrospectively investigated, the APS-associated clinical and laboratory characteristics of female patients stratified by different cut-offs of DRVVT ratio were compared.

**Results:**

The DRVVT NRs of females were significantly lower than those of males on each system. The female patients with DRVVT NR between female-specific and regardless of sex cut-offs had higher positive rates of silica clotting time test and LA retest results after 12 weeks than those with DRVVT NRs lower than female-specific cut-off, there were also more patients who met the APS clinical criteria.

**Conclusion:**

The sex difference of the cut-off value for DRVVT LA test is confirmed on multiple systems, the female-specific cut-off is lower than regardless of sex cut-off and may lead to more female patients being considered as high-risk population for APS.

## Essentials

• A significant difference between the sexes has been found for LA results.
• Three detection systems were enrolled to confirm the sex difference in LA results.
• The LA ratios of females were significantly lower than those of males on each system.
• Female-specific cut-off may lead to more female patients being considered “positive” for LA.

## 1 Introduction

Lupus anticoagulant (LA) testing has been included in the laboratory criteria for the identification of antiphospholipid syndrome (APS) and systemic lupus erythematosus (SLE), respectively [1,2]. The interpretation of LA test has direct bearing on the treatment of these diseases. According to the LA guidance, the International Society on Thrombosis and Haemostasis released in 2020 [3], results are suggestive for LA if LA ratio (screen/confirm) is above the 99th centile of at least 120 healthy donors regardless of sex. However, in a recent study [4], a statistically significant difference between the sexes could be found for the 99th percentile for LA ratio of dilute Russell’s viper venom time (DRVVT) after excluding outliers, and that women had lower cut-off values for LA tests. Here, we attempted to clarify whether this difference exists in various commonly used LA detection systems and the necessity of setting sex-specific cut-off values.

## 2 Methods

### 2.1 Establishing the cut-offs of DRVVT LA test on 3 detection systems

The ACL TOP750 (Werfen), STA-R MAX (Diagnostica Stago), and CS5100 (Sysmex) analyzers with original DRVVT reagents in 3 clinical laboratories (located in Tongji hospital, Qinghai Red Cross hospital, and Qingyuan hospital, respectively) were used in this study. Blood samples from >240 healthy donors (at least 120 cases of each sex, aged between 18 and 60 years) were collected and detected on each system. The samples were double centrifuged at 2000 *g* for 10 minutes to remove platelets, and the platelet-poor plasmas were tested immediately or stored at −70 °C and thawed in a 37 °C water bath before testing.

The DRVVT screen and confirm tests of healthy donors were performed simultaneously (integrated test) on each system. The mixing test of healthy donors was not performed in this study. For each run, patient screen or confirm results (in seconds) were divided by the results of pooled normal plasma (PNP) from 40 healthy adults (in seconds) or commercialized PNP provided by the Zhongchi Weiye Company (China) to obtain the screen (SR) or confirm ratio (CR) [3]. The normalized ratio (NR), expressed as SR/CR, was the final result of LA assay. The sex-specific cut-offs of DRVVT test were obtained based on the 99th or 97.5th centile of SR, CR, and NR grouped by sex in each system [5]. The followed algorithm was used for the identification of outliers before cut-off calculation: outlier if the absolute difference between the index observation (large or small) and the next (largest or smallest) observation was larger than one-third of the range of all observations including the extremes [6].

### 2.2 Evaluation the necessity of setting sex-specific cut-offs for DRVVT test

Female patients admitted to Tongji hospital from October 2023 to May 2024 who were tested for antiphospholipid antibodies due to suspected APS were retrospectively investigated, and their APS-associated clinical events were recorded. Exclusion criteria were age <18 years, LA were detected while receiving anticoagulant treatment (heparin within 24 hours, direct oral or parenteral anticoagulant within 48 hours or warfarin within 7 days), DRVVT results were judged by mixed SR, CR, and NR, and incomplete clinical information.

In the laboratory of Tongji hospital, the screen and confirm tests of LA assay based on DRVVT and silica clotting time (SCT) were performed simultaneously (integrated test) on ACL TOP750 analyzer with original reagents, if screen or confirm tests of LA assay were prolonged, the corresponding mixing test would be performed. The LA results interpretation followed the International Society on Thrombosis and Haemostasis guidance [3]. The IgG and IgM types of anticardiolipin antibody (aCL) and anti-β2GPI antibody (aβ2GpI) were detected by AcuStar chemiluminescence analyzer and original reagents (Werfen). If the female patients could be divided into several groups by female-specific and regardless of sex cut-offs of NR for DRVVT, then the positive rates of SCT, ACA, and Aβ2GpI, LA retest results after 12 weeks, and the incidence of APS-associated clinical events defined in 2023 American College of Rheumatology (ACR)/European League Against Rheumatism (EULAR) APS classification criteria [7] among groups were compared.

Normally and abnormally distributed quantitative variables were compared using the Student's *t*-test and the Mann–Whitney U*-*test, respectively. The chi-squared test is used for the comparison of categorical variable. A *P* value of <.05 is considered statistically significant, and the data analysis was conducted using SPSS 21.0 software. This study was approved by the Ethics Committee of Tongji Medical College, Huazhong University of Science and Technology (2022-S025).

## 3 Results

There were 324 (195 females and 129 males), 243 (122 females and 121 males), and 242 (122 females and 120 males) samples from healthy donors collected and detected on Werfen, Sysmex and Stago systems for DRVVT test, respectively. The SR, CR, and NR between males and females on each system were compared (Figure and Table 1). The SR and NR of females were significantly lower than those of males on each system (*P* < .05).

**Figure:**
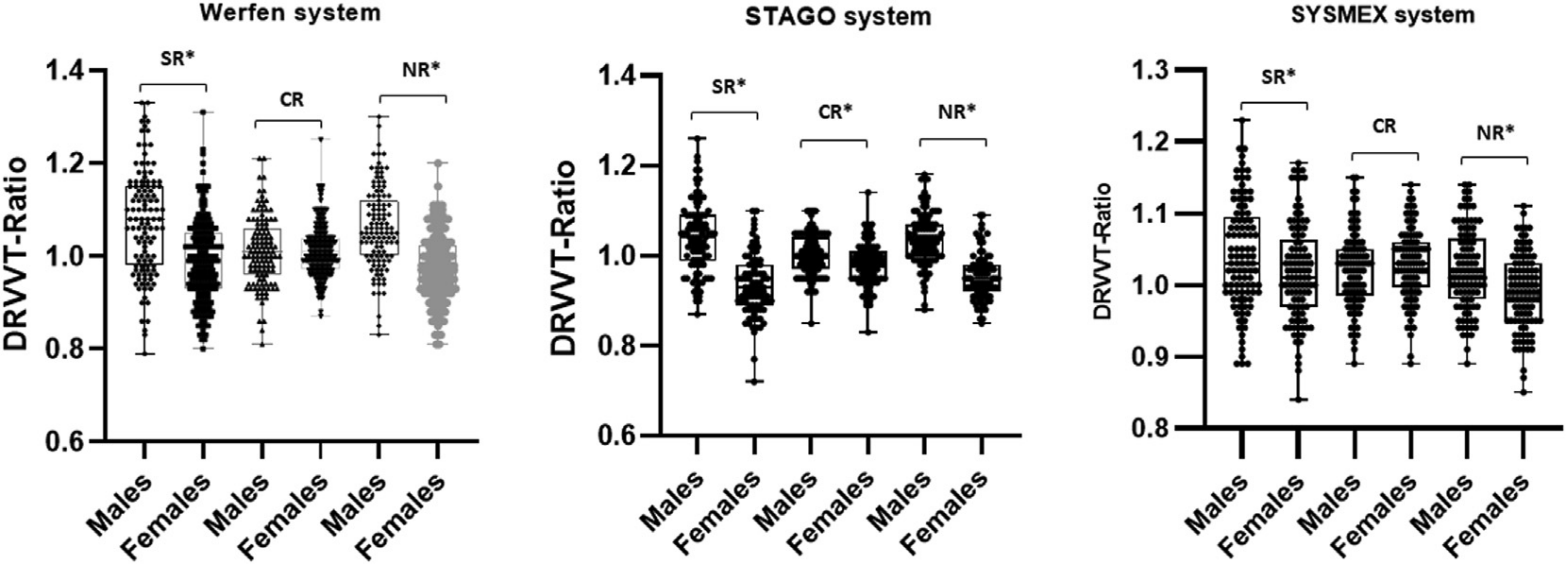
Comparison of dilute Russell’s viper venom time ratios between male and female healthy donors on 3 detection systems. ∗*P* < .05. SR, CR and NR refer to screen ratio, confirm ratio and normalized ratio of dilute Russell’s viper venom time, respectively. Patient screen and confirm results (in seconds) were divided by the corresponding results of pooled normal plasma (in seconds) to obtain the SR and CR, and NR = SR/CR, an NR >cut-off indicates the presence of lupus anticoagulant.

**Table 1:** The cut-offs of dilute Russell’s viper venom time ratios grouped by sex based on 99th or 97.5th centile of healthy donors.

Parameters	DRVVT	ALL	Males	Females	*P* value
Werfen system:		*n* = 324	*n* = 129	*n* = 195	
Ages	Mean±SD	35.6 ± 12.0	36.6 ± 12.8	35.0 ± 11.5	.254
SR	Mean±SD	1.02 ± 0.11	1.07 ± 0.11	0.99 ± 0.09	<.001
	99th centile	1.30	1.32	1.23	
	97.5th centile	1.27	1.29	1.17	
CR	Mean±SD	1.01 ± 0.06	1.01 ± 0.08	1.01 ± 0.06	.941
	99th centile	1.17	1.2	1.15	
	97.5th centile	1.14	1.16	1.14	
NR	Mean±SD	1.01 ± 0.09	1.06 ± 0.09	0.98 ± 0.07	<.001
	99th centile	1.22	1.26	1.14	
	97.5th centile	1.20	1.22	1.11	
Stago system:		*n* = 242	*n* = 120	*n* = 122	
Ages	Mean±SD	38.3 ± 6.6	37.6 ± 6.2	39.2 ± 7.1	.202
SR	Mean±SD	0.99 ± 0.09	1.04 ± 0.08	0.93 ± 0.07	<.001
	99th centile	1.20	1.22	1.10	
	97.5th centile	1.17	1.19	1.09	
CR	Mean±SD	0.99 ± 0.05	1.00 ± 0.05	0.98 ± 0.05	.003
	99th centile	1.10	1.10	1.09	
	97.5th centile	1.08	1.10	1.07	
NR	Mean±SD	1.00 ± 0.07	1.04 ± 0.06	0.95 ± 0.05	<.001
	99th centile	1.17	1.17	1.09	
	97.5th centile	1.14	1.17	1.07	
Sysmex system:		*n* = 243	*n* = 121	*n* = 122	
Ages	Mean±SD	40.4 ± 12.8	42.9 ± 12.9	37.8 ± 12.2	.173
SR	Mean±SD	1.03 ± 0.07	1.04 ± 0.08	1.02 ± 0.07	.019
	99th centile	1.19	1.19	1.16	
	97.5th centile	1.17	1.19	1.16	
CR	Mean±SD	1.03 ± 0.05	1.02 ± 0.05	1.03 ± 0.05	.282
	99th centile	1.14	1.15	1.13	
	97.5th centile	1.13	1.13	1.12	
NR	Mean±SD	1.01 ± 0.06	1.02 ± 0.06	0.99 ± 0.05	<.001
	99th centile	1.13	1.14	1.10	
	97.5th centile	1.12	1.13	1.08	

SR, CR and NR refer to the screen ratio, confirm ratio and normalized ratio of dilute Russell's viper venom time, respectively.

CR, confirm ratio; DRVVT NR, dilute Russell’s viper venom time normalized ratio; NR, normalized ratio; SR, screen ratio.

The mean value, 99th and 97.5th centile of DRVVT SR, CR, and NR grouped by sex in each system are shown in Table 1, respectively. Compared with males, the difference between cut-offs of NR for females and regardless of sex was greater in each system, and considering that the incidence of APS and SLE in females is significantly higher than that in males, we then focused on evaluating the necessity of female-specific cut-off for DRVVT test.Totally 1121 female patients (average age: 38.1±16.3 years) with suspected APS were investigated to evaluate the value of female-specific LA cut-off on Werfen system. The results of DRVVT NR were stratified into positive, gray zone, and negative based on female-specific and regardless of sex cut-offs, and the clinical and laboratory characteristics related to APS among patients with these 3 results were compared (Table 2).

**Table 2:** Comparison of clinical and laboratory characteristics of female patients stratified by different cut-offs of dilute Russell’s viper venom time normalized ratio on Werfen system.

DRVVT NR	Number of patients	Positive SCT	Positive aCL^a^	Positive aβ2GpI^a^	Positive LA retest^b^	Meeting APS clinical criteria^c^
Based on 99th centile						
≥1.22 (Positive)	117	58.1%^d^ (68)	35.0%^d^ (41)	35.9%^d^ (42)	88.9%^d^ (40/45)	58.1% (68)
1.14-1.21 (Gray zone)	128	23.4% (30)	7.8% (10)	7.8% (10)	54.0% (34/63)	52.3% (67)
<1.14 (Negative)	876	11.8%^d^ (103)	6.8% (60)	5.4% (47)	22.5%^d^ (65/289)	26.6%^d^ (233)
Based on 97.5th centile						
≥1.20 (Positive)	148	51.4%^d^ (76)	31.1%^d^ (46)	29.7%^d^ (44)	86.7%^d^ (52/60)	58.8% (87)
1.11-1.19 (Gray zone)	190	23.2% (44)	7.9% (15)	6.3% (12)	45.2% (47/104)	53.7% (102)
<1.11 (Negative)	783	10.3%^d^ (81)	6.4% (50)	5.5% (43)	17.2%^d^ (40/233)	22.9%^d^ (179)

DRVVT NR, dilute Russell’s viper venom time normalized ratio; SCT, silica clotting time; aCL, anticardiolipin antibody; aβ2GpI, anti-β2GP1 antibody; LA, lupus anticoagulant; APS, antiphospholipid syndrome.

a Moderate or high positive of IgG and/or IgM.

b Retest DRVVT or SCT positive after 12 weeks.

c ≥3 points from clinical domains of 2023 ACR/EULAR antiphospholipid syndrome classification criteria.

d *P* < .05 compared with gray zone.

## 4 Discussion

Whether to set sex-specific cut-off value or not has never been mentioned in current LA guidelines [2,3,5]. Only one previous study described the sex difference in LA testing [4], to validate this finding, we performed DRVVT test in healthy blood donors on 3 commonly used detection systems, respectively. The sex difference for LA ratio was confirmed on each system in our study, the cut-off value (99th or 97.5th percentile) of NR for females on each system was lower than that for males.

A previous review found higher values for plasma phospholipids in females than males [8]. In addition, higher levels of coagulation factor (F)II, FVII, FX, FIX, FXI, and FXII have also been found in females as compared with males [9]. These may explain the significantly lower SR for healthy females than males on all 3 systems in our study, and there was no significant difference in CR on 2 of them. Compared with males, the difference between cut-off values of NR for females and for regardless of sex was greater in this study, furthermore, according to previous epidemiologic studies, there are significantly more females than males in patients with autoimmune diseases (ratios of females to males are about 4:1 for APS and 9:1 for SLE) [[10], [11], [12]]. Hence, it can be inferred that a considerable proportion of female patients with suspected APS has LA results that fall between the sex-specific and regardless of sex cut-offs, it seems to be of great significance whether these patients should be considered LA positive or negative. No healthy transgender donors were included in this study, as emerging evidence indicates gender-affirming hormone therapy in transmen modulates immune responses [13]; therefore, future research on transgender populations may help understand the impact of hormones on sex differences in LA results.

In our study, the female patients with gray zone DRVVT result had higher positive rates of SCT test and LA retest after 12 weeks (whether DRVVT or SCT) than those with negative DRVVT result, and also more patients in this group met the APS clinical criteria. The gray zone results, which were traditionally considered negative, may indicate a higher risk of APS than “true” negative results. However, there were also significant differences in the positive rates of SCT, aCL, aβ2GpI, and LA retest between patients with gray zone and positive DRVVT results. Therefore, it is still uncertain whether the gray zone result can be considered as “true” positive.

Both of fresh and frozen samples were used in this study, as the former having the largest proportion, and the difference of DRVVT results between fresh and frozen samples after double centrifuges has been confirmed to be very small [14], it should not have influenced the conclusion of this study. In addition, it is truly difficult to collect normal samples from 120 males and 120 females to obtain the sex-specific cut-offs of LA tests in routine laboratory, this work can be carried out by the reagent manufacturers and just verified with a small number of samples in routine laboratory.

The main limitations of this study include the following: the gender difference was only evaluated for DRVVT test, the LA test based on activated partial thromboplastin time (aPTT), such as SCT, has not been evaluated, due to the different contact activators of aPTT reagents, the sex difference of various LA tests based on aPTT may be heterogenous [15]. Second, the sex difference of LA ratio on a mixture of 1:1 PNP and patient plasma has not been evaluated, as integrated LA test with “paired” performance of the screening and confirmation step are widely used in routine practice [3,16,17], only a few LA results need to be judged based on mix samples. Third, less than half of all the female patients received LA retest after 12 weeks, as it can be imagined that patients who test positive for the first time will be more inclined to come for retest, there may be selection bias when comparing the positive rate of LA retest. Finally, the necessity of setting female-specific cut-offs for DRVVT test was just evaluated by a single-center, retrospective, observational study, the conclusion needs to be confirmed by prospective intervention studies.

In conclusion, the gender difference of the cut-off value for DRVVT LA test is confirmed on multiple detection systems in our study. The female-specific cut-off may lead to more female patients being considered as high-risk population for APS, and further studies are warranted to investigate whether intervention for such patients can bring them benefits.
