# Supplementary material for: Sex-specific cut-off for dilute Russell’s viper venom time lupus anticoagulant test may be of value
Source: Res Pract Thromb Haemost. 2024 Dec 12;9(1):102657. doi: 10.1016/j.rpth.2024.102657 (PMC11741938; doi:10.1016/j.rpth.2024.102657)
Supplement: Supplementary material [file mmc1.docx]

|  | Reagents | Instrument | Lab location |
| --- | --- | --- | --- |
| Werfen system | HemosIL dRVVT Screen and HemosIL dRVVT Confirm;  HemosIL Silica Clotting Time; | ACL TOP750 coagulation analyzer | Tongji hospital, China |
| Stago system | STA-Staclot DRVV Screen and STA-Staclot DRVV Confirm | STA-R MAX coagulation analyzer | Qinghai Red Cross hospital, China |
| Sysmex system | LA1 Screening Reagent and LA2 Confirmation Reagent | CS5100 coagulation analyzer | Qingyuan hospital, China |

**Table1. The list of reagents and instruments for Lupus anticoagulant tests in this study**
